# A reaction network scheme for hidden Markov model parameter learning

**DOI:** 10.1098/rsif.2022.0877

**Published:** 2023-06-21

**Authors:** Carsten Wiuf, Abhishek Behera, Abhinav Singh, Manoj Gopalkrishnan

**Affiliations:** ^1^ Department of Mathematical Sciences, University of Copenhagen, Copenhagen, Denmark; ^2^ Department of Electrical Engineering, Indian Institute of Technology Bombay, Mumbai, India; ^3^ UM-DAE Centre for Excellence in Basic Sciences, Mumbai, India

**Keywords:** molecular programming, synthetic biology, hidden Markov model, Baum–Welch algorithm, statistical learning

## Abstract

With a view towards artificial cells, molecular communication systems, molecular multiagent systems and federated learning, we propose a novel reaction network scheme (termed the Baum–Welch (BW) reaction network) that learns parameters for hidden Markov models (HMMs). All variables including inputs and outputs are encoded by separate species. Each reaction in the scheme changes only one molecule of one species to one molecule of another. The reverse change is also accessible but via a different set of enzymes, in a design reminiscent of futile cycles in biochemical pathways. We show that every positive fixed point of the BW algorithm for HMMs is a fixed point of the reaction network scheme, and vice versa. Furthermore, we prove that the ‘expectation’ step and the ‘maximization’ step of the reaction network separately converge exponentially fast and compute the same values as the E-step and the M-step of the BW algorithm. We simulate example sequences, and show that our reaction network learns the same parameters for the HMM as the BW algorithm, and that the log-likelihood increases continuously along the trajectory of the reaction network.

## 1. Introduction

The implementation of abstract dynamical systems with molecular systems has gained scientific interest as a promising piece in the nanotechnology toolbox. Several automated theoretical schemes can now compile arbitrary networks of formal reactions into DNA oligonucleotide sequences [1–8]. Experimental demonstrations have synthesized these oligonucleotides, mixed them in a single test tube, and verified that they interact via base pairing reactions to implement the dynamics of the formal network [2,7,9–11]. In this way, if an algorithm can be described by reaction network dynamics, it might equally be carried out in a test tube by a DNA machine. Understanding how to describe algorithms in terms of reaction network dynamics is thus becoming a matter of interest.

Dynamical systems described by formal reaction networks are known to be computationally universal [12]. Several examples of functions computed by reaction network dynamics have been described [13–20]. However, the computational universality proofs often do not lend to the most elegant ways of implementing the algorithms with chemical reaction networks. We are particularly interested in problems arising in statistical learning theory, such as maximum-likelihood estimation and related optimization problems, Bayesian posterior sampling, and inference from incomplete data. These problems are at the core of statistical theory and may have many practical applications when implemented inside artificial cells. Our work demonstrates that these problems are particularly amenable to implementation by reaction network dynamics by exploiting the formal connection between the notion of entropy in statistics and in statistical mechanics.

Molecule-based statistical inference is receiving increasing attention. In [21], dynamic Bayesian decision-making in the form of a two-state hidden Markov model (HMM) is implemented by means of intracellular kinetics (which might be interpreted in terms of reaction networks), where the target is to infer an approximate posterior distribution. Similar ideas have been applied to decode information in biological processes [22]. On a different note, a cell’s potential for solving tasks and learning requires a thorough understanding of design principles in reaction networks and how molecular sources of stochastic information are communicated within and between cells [23,24]. Such tasks might be seen as computational and statistical problems posed to the cell.

In this article, we describe a reaction network scheme whose dynamics learns parameters of HMMs. HMMs are standard statistical models widely used in machine learning to model complex data with a linear spatial structure as in bioinformatics [25], or a temporal structure as in speech recognition [26]. HMMs also form an essential component of communication systems as well as of intelligent agents trained by reinforcement learning methods. They might be used in an exploratory sense without stipulating the interpretation of the hidden variables in advance, or in a concrete sense to learn the strength and influence of known hidden variables. Our group has previously worked on statistical learning theory from the perspective of reaction networks and log-linear models [27–31], and the current paper builds on these experiences.

Our proposed algorithm has similarities to, and important differences from, the Baum–Welch (BW) algorithm [32] which is the standard learning algorithm for HMMs. The BW algorithm is an iterative expectation-maximization (EM) algorithm where one step is performed at a time in a prescribed sequence. Our reaction network scheme is divided into four subnetworks that correspond to the forward algorithm, the backward algorithm, the expectation step (E-step), and the maximization step (M-step) of the BW algorithm. Each subnetwork describes a system of ordinary differential equations (ODEs) that might be run separately, exactly mimicking the steps of the BW algorithm; or run simultaneously in continuous time and in a distributed manner, obtaining a variant on the BW theme where all four stages of the BW algorithm are being performed at the same time. Because our algorithm permits the different stages to be run simultaneously and without coordination, it is particularly suited to software federated learning implementations where HMMs might need to be run on a network of edge devices in a distributed manner.

We obtain the following results.

— Our scheme can be partitioned into two modules, the inference module (forward, backward, E-step) and the learning module (M-step), which provide feedback to each other. We show in theorem 4.2 that each module separately converges exponentially fast to the correct value (the value of the BW algorithm) when the other module is kept switched off.
— We prove in theorem 4.3 that our scheme (when divided into two modules, or when considered jointly) and the BW algorithm have the same set of positive fixed points.
— We demonstrate practical feasibility of our algorithm by simulation of example HMMs, and by showing that parameters can be learned successfully with performance comparable to that of the BW algorithm.

The proposed reaction network is a formal (abstract) reaction network without particular chemical features. It might be turned into a chemically realistic reaction network by compiling the formal reactions into reactions between DNA oligonucleotide sequences, using recently proposed techniques [1–4]. Promising areas of application of this work come from cellular biology. In many cellular processes, only partial information about the environment is available in the form of a sequence of observations. For example, this might happen when an enzyme acts processively on a substrate or a molecular walker locates its position on a grid [33–35]. In the future, a molecule-based HMM device might learn a molecular environment within an organism by sensing and interacting with the environment at the molecular level. It might take action according to the learning outcome, for example, choosing among different drug options, or a molecule-based HMM might be used as a building block in an artificial cell or population of cells, enabling cooperative behaviour among cells or facilitating various tasks.

## 2. The Baum–Welch algorithm

A **stochastic map** between two finite sets *P* and *Q* is a matrix *A* = (*a*_*pq*_)_*P*×*Q*_, such that *a*_*pq*_ ≥ 0 for *p* ∈ *P*, *q* ∈ *Q*, and $\sum_{q\in Q}a_{\;pq}=1$ for *p* ∈ *P*. One might think of a stochastic map as a collection of conditional probability distributions.

**Figure 1.**
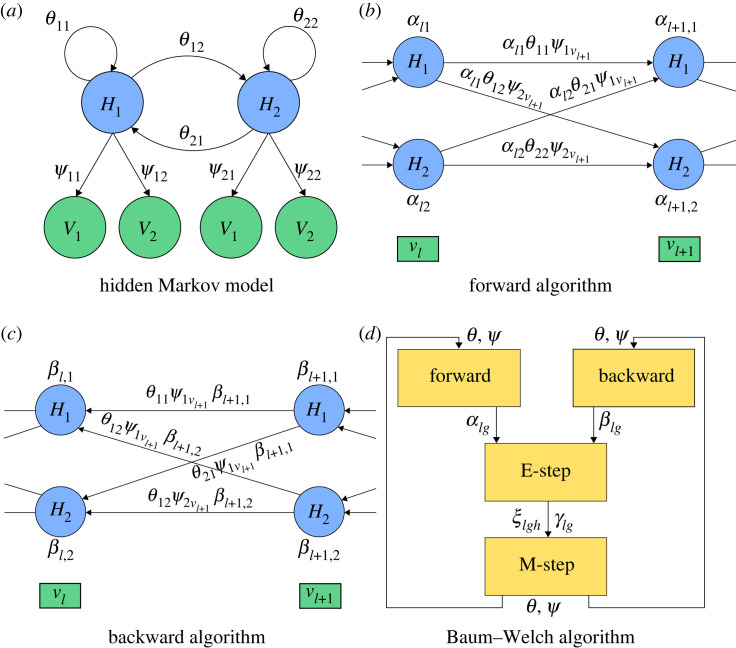
Learning HMMs from sequences. (*a*) **HMM**: the hidden states, elements of *H* = {*H*_1_, *H*_2_}, are not directly observable. Instead, we observe elements from a set *V* = {*V*_1_, *V*_2_} of visible states. The parameters *θ*_11_, *θ*_12_, *θ*_21_, *θ*_22_ denote the probability of transitions between the hidden states. The probability of observing the states *V*_1_, *V*_2_ depends on the parameters *ψ*_11_, *ψ*_12_, *ψ*_21_, *ψ*_22_, as indicated in the figure. (*b*) The **forward algorithm** computes the likelihood of the first ℓ + 1 observed states (the position ℓ + 1 likelihood) $\alpha_{\ell +1,1}=\alpha_{\ell 1}\theta_{11}\psi_{1v_{\ell +1}}+\alpha_{\ell 2}\theta_{21}\psi_{1v_{\ell +1}}$ by forward propagating the position ℓ likelihoods *α*_ℓ1_ and *α*_ℓ2_. Here, *v*_ℓ_, *v*_ℓ+1_ ∈ *V* are the observed symbols at position ℓ and ℓ + 1, respectively. (*c*) The **backward algorithm** computes the conditional probability of the observed states ℓ + 1, …, *L*, given the observed state ℓ (the position ℓ conditional probability) $\beta_{\ell 1}=\theta_{11}\psi_{1v_{\ell +1}}\beta_{\ell +1,1}+\theta_{12}\psi_{2v_{\ell +1}}\beta_{\ell +1,2}$ by propagating the position ℓ conditional probabilities *β*_ℓ+1,1_ and *β*_ℓ+1,2_ backwards. (*d*) The **BW algorithm** is a fixed point expectation-maximization computation. The E-step calls the forward and backward algorithm as subroutines and, conditioned on the entire observed sequence $(v_{1},v_{2},\ldots ,v_{L})\in V^{L}$, computes the probabilities *γ*_ℓ*g*_ of being in states *g* ∈ *H* at position ℓ and the probabilities *ξ*_ℓ*gh*_ of taking the transitions (*g*, *h*) ∈ *H*^2^ at position ℓ. The M-step updates the parameters *θ* and *ψ* to maximize their likelihood given the observed sequence.

An **HMM** is a tuple (*H*, *V*, *θ*, *ψ*, *π*) of two finite sets *H* (for ‘hidden’) and *V* (for ‘visible’), an **initial probability distribution** *π* = (*π*_*h*_)_*h*∈*H*_ on *H*, and two stochastic maps: the **transition matrix** *θ* : *H* → *H*, and the **emission matrix** *ψ* : *H* → *V*. See figure 1 for an example.

The **likelihood** $P(v_{1},\ldots ,v_{L}|\theta ,\psi )$ of an observed sequence *v*_1_, …, *v*_*L*_ ∈ *V* given the parameter (*θ*, *ψ*) is the probability

2.1 $$P(v_{1},\ldots ,v_{L}|\theta ,\psi )=\underset{\eta \in H^{L}}{\sum }\;\pi_{h_{1}}\psi_{h_{1}v_{1}}\overset{L}{\underset{\ell =2}{\prod }}\theta_{h_{\ell -1}h_{\ell }}\psi_{h_{\ell }v_{\ell }},$$

where the sum is over all sequences *η* = (*h*_1_, …, *h*_*L*_) ∈ *H*^*L*^, with *L* ≥ 2 (to avoid triviality). Knowing *π* and the sequence $\left(v_{1},v_{2},\ldots ,v_{L}\right)$ of visible states, one can estimate maximum-likelihood values for (*θ*, *ψ*) by means of the BW algorithm, which is a particular instance of the EM algorithm. In §5, we show how to extend this construction to unknown *π* and multiple sequences while preserving the theoretical guarantees.

The standard BW algorithm is composed of four subroutines. The **forward algorithm** (figure 1*b*) outputs the quantities *α*_ℓ*h*_ computed from initial values of (*θ*, *ψ*) and *π* by the recursion

$$\alpha_{1h}=\pi_{h}\psi_{hv_{1}}\;\mathrm{and}\;\alpha_{\ell +1,h}=\sum_{g\in H}\alpha_{\ell g}\theta_{gh}\psi_{hv_{\ell +1}},$$

and the **backward algorithm** (figure 1*c*) outputs the quantities *β*_ℓ*h*_ computed by the recursion

$$\beta_{Lh}=1\;\mathrm{and}\;\beta_{\ell h}=\sum_{g\in H}\theta_{hg}\psi_{gv_{\ell +1}}\beta_{\ell +1,g},$$

where *h* ∈ *H* and ℓ = 1, …, *L* − 1. The **E-step** computes

$$\xi_{\ell gh}=\frac{\alpha_{\ell g}\theta_{gh}\psi_{hv_{\ell +1}}\beta_{\ell +1,h}}{\sum_{\;f\in H}\alpha_{\ell f}\beta_{\ell f}}\;\mathrm{and}\;\gamma_{\ell h}=\frac{\alpha_{\ell h}\beta_{\ell h}}{\sum_{\;f\in H}\alpha_{\ell f}\beta_{\ell f}},$$

for ℓ ∈ {1, 2, …, *L*} and *g*, *h* ∈ *H*. Finally, the **M-step** computes an update of (*θ*, *ψ*),

$$\theta_{gh}^{\prime }=\frac{\sum_{\ell =1}^{L-1}\xi_{\ell gh}}{\sum_{\ell =1}^{L-1}\sum_{\;f\in H}\xi_{\ell gf}}\;\mathrm{and}\;\psi_{hw}^{\prime }=\frac{\sum_{\ell =1}^{L}\gamma_{\ell h}\delta_{w,v_{\ell }}}{\sum_{\ell =1}^{L}\gamma_{\ell h}},$$

for *g*, *h* ∈ *H* and *w* ∈ *V*, where *δ* is the Dirac delta function $\delta_{w,v_{\ell }}=1$ iff *w* = *v*_ℓ_. Here, *θ*_*gh*_′ and *ψ*_*hw*_′ (with primes) are the updated parameter values of *θ*_*gh*_ and *ψ*_*hw*_, respectively, after one iteration of the BW algorithm. As *π* is assumed known, it is not updated.

The BW algorithm (figure 1*d*) sequentially runs the forward algorithm, the backward algorithm, the E-step, and the M-step until the change in the likelihood (2.1) is insignificant. In practice, this means until the difference between (*θ*, *ψ*) and (*θ*′, *ψ*′) becomes smaller than a prescribed tolerance level. For the steps to be well defined, division by zero is not allowed. Denote the parameter region for which division by zero does not happen in any step of an iteration by

$$\Theta =\{(\theta ,\psi )\;:\;\overset{L}{\underset{\ell =1}{\sum }}\alpha_{\ell h}\beta_{\ell h}>0,\;h\in H\}$$

(see the electronic supplementary material for details and proof). Furthermore, let $\Theta_{0}=\{(\theta ,\psi )\;|\;\theta >0,\psi >0\}$ and $\Theta_{1}=\{(\theta ,\psi )|\;\theta \geq 0,\psi \geq 0\}$ (the full parameter space), where vector/matrix inequalities are taken coordinate-wise. It follows that $\Theta_{0}\subseteq \Theta \subseteq \Theta_{1}$, and that all steps in one iteration of the BW algorithm are valid, provided (θ,ψ)∈Θ. Each row (a conditional distribution) of the update (*θ*′, *ψ*′) has unit length automatically.

The following lemma is essential. See the electronic supplementary material for a proof.

**Lemma 2.1.**

*Assume all letters of* *V* *are in the observed sequence* *v*_1_, …, *v*_*L*_ *and* *π* > 0, *L* ≥ 2, *or all letters of* *V* are in *v*_2_, …, *v*_*L*_ (excluding *v*_1_) and *π* ≥ 0, *L* ≥ 3. *If* $(\theta ,\psi )\in \Theta_{0}$, *then the same holds for the updated parameter value*, $(\theta^{\prime },\psi^{\prime })\in \Theta_{0}$.

Let (*θ*_*n*_, *ψ*_*n*_) denote the value of (*θ*′, *ψ*′) after *n* iterations of the BW algorithm. According to lemma 2.1, if $(\theta_{0},\psi_{0})\in \Theta_{0}$, then $(\theta_{n},\psi_{n})\in \Theta_{0}$ for all *n* ≥ 0. If $(\theta_{n},\psi_{n})\to (\theta^{*},\psi^{*})\in \Theta_{0}$ as *n* → ∞, then it is a local extremum or saddle point of the likelihood (2.1) [36–39]. If $(\theta^{*},\psi^{*})\in \Theta$, then it is a fixed point of the BW algorithm. However, the limit might be outside Θ [36–38]. In general, the BW algorithm might have multiple fixed points depending on the initial point (*θ*_0_, *ψ*_0_) and the observed sequence.

Henceforth, we make the assumptions of lemma 2.1. If *v* ∈ *V* is not observed, then *ψ*_*hv*_ = 0 for all *h* ∈ *H*, and one might equivalently consider a HMM with the state *v* removed from *V*. Hence, the real (mild) restriction is *π* > 0, *L* ≥ 2, or *π* ≥ 0, *L* ≥ 3.

## 3. Baum–Welch reaction network

Let *S* be a finite set. A formal (chemical) reaction over *S* is a pair $a,b\in Z_{\geq 0}^{S}$ of non-negative integer vectors. This is commonly represented in chemical equation notation as $\sum_{i\in S}a_{i}X_{i}\to \sum_{i\in S}b_{i}X_{i}$, where *X*_*i*_, *i* ∈ *S*, represent formal (chemical) species. Given a rate constant *k* > 0, **mass-action kinetics** describes the change of concentrations through time by the system of ODEs $\dot{X}(t)=(b-a)k\prod_{i\in S}X_{i}{\left(t\right)}^{a_{i}}$, where species names are overloaded to also represent the vector of concentrations *X*(*t*) = (*X*_*i*_(*t*))_*i*∈*S*_ at time *t* ≥ 0. A **reaction network** is a finite collection (*a*_1_, *b*_1_), (*a*_2_, *b*_2_), $\ldots ,(a_{m},b_{m})$ of formal reactions, together with a choice of reaction rate constants. Combining the effect of all reactions yields the ODE system $\dot{X}(t)=\sum_{\;j=1}^{m}(b_{j}-a_{j})k_{j}\prod_{i\in S}X_{i}{\left(t\right)}^{a_{\;ji}}$. For background on reaction networks, see [40].

We now describe a reaction network that implements HMM learning. Our scheme bears close correspondence to the BW algorithm as presented in the previous section. Let an HMM (*H*, *V*, *θ*, *ψ*, *π*) and an observed sequence $(v_{1},v_{2},\ldots ,v_{L})\in V^{L}$ be given. Choose an arbitrary hidden state h* ∈ *H* and an arbitrary visible state v* ∈ *V*. This choice is merely an artifice to break symmetry, and our results hold independently of these choices. We represent every variable appearing in the BW algorithm by a separate species. The species are *θ*_*gh*_, *θ*_*gh*_′, *ψ*_*hw*_, *ψ*_*hw*_′, *π*_*h*_, *α*_ℓ*h*_, *β*_ℓ*h*_, *γ*_ℓ*h*_, *ξ*_ℓ*gh*_ with indices *g*, *h* ∈ *H*, *w* ∈ *V*, ℓ = 1, …, *L*. That is, both (*θ*, *ψ*) and the update (*θ*′, *ψ*′) are represented as species.

We first work out in full detail how the forward algorithm may be translated into chemical reactions. Recall that the forward algorithm is the recursion

$$\alpha_{1h}=\pi_{h}\psi_{hv_{1}}\;\mathrm{and}\;\alpha_{\ell +1,h}=\sum_{g\in H}\alpha_{\ell g}\theta_{gh}\psi_{hv_{\ell +1}},$$

for ℓ = 1, …, *L* − 1 and *g*, *h* ∈ *H*. This implies the balance equations

3.1 $$\alpha_{1h}\pi_{h^{*}}\psi_{h^{*}v_{1}}=\alpha_{1h^{*}}\pi_{h}\psi_{hv_{1}}$$

and

3.2 $$\alpha_{\ell +1,h}\sum_{g\in H}\alpha_{\ell g}\theta_{gh^{*}}\psi_{h^{*}v_{\ell +1}}=\alpha_{\ell +1,h^{*}}\sum_{g\in H}\alpha_{\ell g}\theta_{gh}\psi_{hv_{\ell +1}},$$

for all *h* ∈ *H* and ℓ = 1, …, *L* − 1. For the initialization step, this prompts the use of the reactions

$$\alpha_{1h}+\pi_{h^{*}}+\psi_{h^{*}v_{1}}\overset{1}{\to }\alpha_{1h^{*}}+\pi_{h^{*}}+\psi_{h^{*}v_{1}}$$

and

$$\alpha_{1h^{*}}+\pi_{h}+\psi_{hv_{1}}\overset{1}{\to }\alpha_{1h}+\pi_{h}+\psi_{hv_{1}},$$

for all *h* ∈ *H*, where $\overset{1}{\to }$ indicates the reaction rate constant is put to 1. Only one species change in each reaction (the red species), the other species are **catalysts** that remain unchanged by the reaction. The rate by which *α*_1*h*_ is converted into *α*_1h*_ depends on the concentrations of the catalysts and, thus, is time-dependent. By design, at equilibrium the set of reactions fulfil the balance equation (3.1). For the recursion step, we use the reactions

$$\begin{array}{rl} & \alpha_{\ell +1,h}+\alpha_{\ell g}+\theta_{gh^{*}}+\psi_{h^{*}v_{\ell +1}}\overset{1}{\to }\alpha_{\ell +1,h^{*}}+\alpha_{\ell g}+\theta_{gh^{*}}+\psi_{h^{*}v_{\ell +1}} \end{array}$$

and

$$\begin{array}{rl} & \alpha_{\ell +1,h^{*}}+\alpha_{\ell g}+\theta_{gh}+\psi_{hv_{\ell +1}}\overset{1}{\to }\alpha_{\ell +1,h}+\alpha_{\ell g}+\theta_{gh}+\psi_{hv_{\ell +1}}, \end{array}$$

for all *g*, *h* ∈ *H*, and ℓ = 1, …, *L* − 1. Again by design, at equilibrium the balance equation (3.2) is fulfilled for this set of reactions.

The reactions depend on the observed sequence $(v_{1},v_{2},\ldots ,v_{L})\in V^{L}$ of visible states. This is a problem because one would have to design different reaction networks for different observed sequences, defeating the whole purpose. To solve this problem, we introduce the catalyst species *E*_ℓ*w*_ with ℓ=1,…,L and *w* ∈ *V*. The *E*_ℓ*w*_ species are initialized such that at time zero, *E*_ℓ*w*_(0) = 1 for *w* = *v*_ℓ_, and *E*_ℓ*w*_(0) = 0 for $w\neq v_{\ell }$. Thus, their concentrations remain fixed throughout time.

The other parts of the BW algorithm may be translated into chemical reactions using a similar logic. The full set of reactions is shown in table 1 and is termed the BW reaction network. It consists of four parts that are further divided into smaller subnetworks. Each of the four parts corresponds to one of the four parts of the BW algorithm, as shown in table 1. The corresponding equations of the ODE system are shown in appendix A (equations (A 1)–(A 8)). Each of the smaller subnetworks in table 1 when run independently can be separately analysed as a mono-molecular reaction network for which the dynamical behaviour can be fully described as shown in appendix A.

**Table 1.** The BW reaction network, divided into four parts corresponding to the forward ($R_{1}^{\alpha },R_{\ell }^{\alpha }$) and the backward ($R_{\ell }^{\beta }$) algorithm, the E-step ($R_{\ell }^{\gamma },R_{\ell }^{\xi }$) and the M-step ($R^{\theta },R^{\psi }$). All parts but the M-step are further divided into small subnetworks, one for each ℓ = 1, …, *L* − 1 (or *L*). Catalytic species are in black, non-catalytic in red. The indices vary over *g*, *h* ∈ *H*, *w* ∈ *V*, and ℓ=1,…,L−1. For *γ* species, ℓ = *L* is also allowed.

BW algorithm	BW reaction network	subnetwork
$\begin{array}{c} \alpha_{1h}=\pi_{h}\psi_{hv_{1}}\;\;\\ \alpha_{\ell +1,h}=\sum_{g\in H}\alpha_{\ell g}\theta_{gh}\psi_{hv_{\ell +1}} \end{array}$	$\begin{array}{rl} & \alpha_{1h}+\pi_{h^{*}}+\psi_{h^{*}w}+E_{1w}\to \alpha_{1h^{*}}+\pi_{h^{*}}+\psi_{h^{*}w}+E_{1w}\\ & \;\alpha_{1h^{*}}+\pi_{h}+\psi_{hw}+E_{1w}\to \alpha_{1h}+\pi_{h}+\psi_{hw}+E_{1w}\\ & \alpha_{\ell +1,h}+\alpha_{\ell g}+\theta_{gh^{*}}+\psi_{h^{*}w}+E_{\ell +1,w}\to \alpha_{\ell +1,h^{*}}+\alpha_{\ell g}+\theta_{gh^{*}}+\psi_{h^{*}w}+E_{\ell +1,w}\\ & \;\alpha_{\ell +1,h^{*}}+\alpha_{\ell g}+\theta_{gh}+\psi_{hw}+E_{\ell +1,w}\to \alpha_{\ell +1,h}+\alpha_{\ell g}+\theta_{gh}+\psi_{hw}+E_{\ell +1,w} \end{array}$	$\begin{array}{c} R_{1}^{\alpha }\\ R_{\ell }^{\alpha } \end{array}$
$\begin{array}{c} \beta_{Lh}=1\;\;\\ \beta_{\ell h}=\sum_{g\in H}\theta_{hg}\psi_{gv_{\ell +1}}\beta_{\ell +1,g} \end{array}$	$\begin{array}{r} \beta_{\ell h}+\beta_{\ell +1,g}+\theta_{h^{*}g}+\psi_{gw}+E_{\ell +1,w}\to \beta_{\ell h^{*}}+\beta_{\ell +1,g}+\theta_{h^{*}g}+\psi_{gw}+E_{\ell +1,w}\\ \beta_{\ell h^{*}}+\beta_{\ell +1,g}+\theta_{hg}+\psi_{gw}+E_{\ell +1,w}\to \beta_{\ell h}+\beta_{\ell +1,g}+\theta_{hg}+\psi_{gw}+E_{\ell +1,w} \end{array}$	$R_{\ell }^{\beta }$
$\begin{array}{rl} & \gamma_{\ell h}=\frac{\alpha_{\ell h}\beta_{\ell h}}{\sum_{g\in H}\alpha_{\ell g}\beta_{\ell g}\;\;}\\ & \xi_{\ell gh}=\frac{\alpha_{\ell g}\theta_{gh}\psi_{hv_{\ell +1}}\beta_{\ell +1,h}}{\sum_{\;f\in H}\alpha_{\ell f}\beta_{\ell f}} \end{array}$	$\begin{array}{rl} & \gamma_{\ell h}+\alpha_{\ell h^{*}}+\beta_{\ell h^{*}}\to \gamma_{\ell h^{*}}+\alpha_{\ell h^{*}}+\beta_{\ell h^{*}}\\ & \;\gamma_{\ell h^{*}}+\alpha_{\ell h}+\beta_{\ell h}\to \gamma_{\ell h}+\alpha_{\ell h}+\beta_{\ell h}\\ & \xi_{\ell gh}+\alpha_{\ell h^{*}}+\theta_{h^{*}h^{*}}+\beta_{\ell +1,h^{*}}+\psi_{h^{*}w}+E_{\ell +1,w}\to \\ & \;\xi_{\ell h^{*}h^{*}}+\alpha_{\ell h^{*}}+\theta_{h^{*}h^{*}}+\beta_{\ell +1,h^{*}}+\psi_{h^{*}w}+E_{\ell +1,w}\\ & \xi_{\ell h^{*}h^{*}}+\alpha_{\ell g}+\theta_{gh}+\beta_{\ell +1,g}+\psi_{hw}+E_{\ell +1,w}\to \\ & \;\xi_{\ell gh}+\alpha_{\ell g}+\theta_{gh}+\beta_{\ell +1,g}+\psi_{hw}+E_{\ell +1,w} \end{array}$	$\begin{array}{c} R_{\ell }^{\gamma }\\ R_{\ell }^{\xi } \end{array}$
$\begin{array}{c} \theta_{gh}^{\prime }=\frac{\sum_{\ell =1}^{L-1}\xi_{\ell gh}}{\sum_{\ell =1}^{L-1}\sum_{\;f\in H}\xi_{\ell gf}}\\ \psi_{hw}^{\prime }=\frac{\sum_{\ell =1}^{L}\gamma_{\ell h}\delta_{w,v_{\ell }}}{\sum_{\ell =1}^{L}\gamma_{\ell h}} \end{array}$	$\begin{array}{c} \theta_{gh}^{\prime }+\xi_{\ell gh^{*}}\to \theta_{gh^{*}}^{\prime }+\xi_{\ell gh^{*}}\\ \;\theta_{gh^{*}}^{\prime }+\xi_{\ell gh}\to \theta_{gh}^{\prime }+\xi_{\ell gh}\\ \psi_{hw}^{\prime }+\gamma_{\ell h}+E_{\ell v^{*}}\to \psi_{hv^{*}}^{\prime }+\gamma_{\ell h}+E_{\ell v^{*}}\\ \;\psi_{hv^{*}}^{\prime }+\gamma_{\ell h}+E_{\ell w}\to \psi_{hw}^{\prime }+\gamma_{\ell h}+E_{\ell w} \end{array}$	$\begin{array}{c} R^{\theta }\\ R^{\psi } \end{array}$

## 4. The dynamics of the Baum–Welch reaction network

In the following, we expose the relationship between the dynamics of the BW algorithm and the BW reaction network. For this, we define three different, alternative ways of running the BW reaction network in table 1.

Let *α*_ℓ_(*t*) = (*α*_ℓ*h*_(*t*))_*h*∈*H*_ denote the vector of concentrations at time *t* ≥ 0, with similar notation for other quantities. For convenience, we leave out the species *E*_ℓ*w*_.

### 4.1. BW1

*Initialization.* Fix an initial value, $(\theta ,\psi )=(\theta_{0},\psi_{0})\in \Theta_{0}$. Initialize the concentrations at time *t* = 0: *α*_ℓ_(0), *β*_ℓ_(0), *γ*_ℓ_(0) $\in R_{\geq 0}^{H}$, *ξ*_ℓ_(0), $\theta^{\prime }(0)\in R_{\geq 0}^{H\times H}$ and $\psi^{\prime }(0)\in R^{H\times V}$, such that $\sum_{h}\alpha_{\ell h}(0)=A_{\ell }$, $\sum_{h}\beta_{\ell h}(0)=B_{\ell }$, $\sum_{h}\gamma_{\ell h}(0)=1$, $\sum_{h}\eta_{\ell gh}(0)=1$, $\sum_{h}\theta_{gh}^{\prime }(0)=1$ and $\sum_{v}\psi_{hv}^{\prime }(0)=1$, for arbitrary positive constants *A*_ℓ_, *B*_ℓ_.

*Execution.* The ODE systems of the subnetworks are executed sequentially in the order $R_{1}^{\alpha },\ldots ,R_{L}^{\alpha }$, $R_{L-1}^{\beta },\ldots ,R_{1}^{\beta }$, $R_{1}^{\gamma },$ $\ldots ,R_{L}^{\gamma }$, $R_{1}^{\xi },\ldots ,R_{L-1}^{\xi }$, $R^{\theta }$, $R^{\psi }$ (table 1), such that the ODE system of the *k*th subnetwork (*k* = 1, …, 4*L* − 1) is run until an equilibrium is obtained with the chosen initial values, before the ODE system of the (*k* + 1)th subnetwork is executed. The catalyst species concentrations of the *k*th subnetwork are fixed to their equilibrium values obtained in the *k*′th subnetworks, *k′* < *k*. In particular, the concentrations of the species *θ*, *ψ* are fixed to their initial values $(\theta_{0},\psi_{0})\in \Theta_{0}$.

*Iteration.* The above procedure is then iterated. After completion of the *n*th iteration, *n* ≥ 0, the concentrations of the species *θ*, *ψ* in the (*n* + 1)th iteration are initialized to the equilibrium values $(\theta_{n},\psi_{n})\in \Theta_{0}$ of the species (*θ*′, *ψ*′) in the *n*th iteration. All other species are initialized at their current values.

*Completion.* This process is continued until convergence of (*θ*_*n*_, *ψ*_*n*_) has been achieved. The limit of (*θ*_*n*_, *ψ*_*n*_) is denoted the equilibrium of BW1 with initial point (*θ*_0_, *ψ*_0_).

### 4.2. BW2

*Initialization.* As in BW1.

*Execution.* The ODE systems corresponding to the inference module (forward, backward and E-step) and the learning module (M-step) of the BW algorithm are executed sequentially and run until an equilibrium is obtained, with the concentrations of the species *θ*, *ψ* fixed to the initial values $(\theta_{0},\psi_{0})\in \Theta_{0}$.

**Figure 2.**
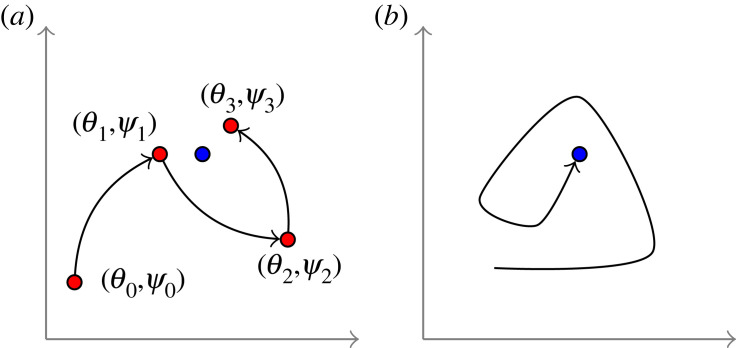
(*a*) Iterations of BW2. In the *n*th iteration, *n* ≥ 0, the concentrations of (*θ*, *ψ*) are fixed at (*θ*_*n*_, *ψ*_*n*_). The ODE system is initialized at (*θ*′(0), *ψ*′(0)) = (*θ*_*n*_, *ψ*_*n*_). Over time (*θ*′(*t*), *ψ*′(*t*)) converges to (*θ*_*n*+1_, *ψ*_*n*+1_). As *n* → ∞, (*θ*_*n*_, *ψ*_*n*_) converges to the blue dot. (*b*) A trajectory of BW3 converging towards the blue dot, initialized at a (random) point in the parameter space.

*Iteration and completion.* BW2 is iterated similarly to BW1. The limit of (*θ*_*n*_, *ψ*_*n*_) is denoted the equilibrium of BW2 with initial point (*θ*_0_, *ψ*_0_). See figure 2.

### 4.3. BW3

*Initialization.* As in BW1 with (*θ*′(0), *ψ*′(0)) = (*θ*_0_, *ψ*_0_).

*Execution.* The species *θ*, *ψ* are identified with the species *θ*′, *ψ*′. That is, unprimed species are substituted for primed ones in table 1. The full ODE system as described in §3 is executed at the same time without fixing any species concentrations so that all species concentrations are dynamic.

*Completion.* The limit of (*θ*′(*t*), *ψ*′(*t*)) (if it exists) is denoted the equilibrium of BW3 with initial point (*θ*_0_, *ψ*_0_).

BW1 replicates the BW algorithm with one equilibrium update for each step in one iteration of the BW algorithm. In total, there are 4*L* steps in one iteration of the BW algorithm, equalling the number of subnetworks (table 1). BW2 combines all 4*L* steps in one iteration of the BW algorithm, corresponding to the simultaneous calculation of the forward and backward algorithm, the E-step and the M-step in one iteration. BW3 makes all updates simultaneously. BW3 is a feedback system where the concentrations of all species are adjusted continuously.

Proofs of the following statements are in appendix A.

**Theorem 4.1.**

*If the BW algorithm and BW1 are initiated at the same point* $(\theta_{0},\psi_{0})\in \Theta_{0}$, *then their equilibria always exist and agree*.

**Theorem 4.2.**

*If BW1 and BW2 are initiated at the same point* $(\theta_{0},\psi_{0})\in \Theta_{0}$, *then their equilibria always exist and agree. Furthermore, this equilibrium is globally asymptotically stable for the BW2 dynamics and convergence is exponentially fast, subject to the invariant subspace defined by the initialization of* *θ*′, *ψ*′: *the sum of the entries of each row of* *θ*′(*t*), *respectively*, *ψ*′(*t*), *is one*.

The two theorems imply that the initial conditions of the species other than (*θ*′, *ψ*′) are irrelevant, hence we need not be concerned with these. Furthermore, BW1 and BW2 compute the same parameter value as the BW algorithm provided $(\theta_{0},\psi_{0})\in \Theta_{0}$. Theorems 4.1 and 4.2 might *not* be true if (*θ*_0_, *ψ*_0_) is at the boundary $\Theta \setminus \Theta_{0}$; see below for discussion. In particular, an equilibrium of BW3 at the boundary might not be an equilibrium of the BW algorithm.

**Theorem 4.3.**

*The sets of positive equilibria of BW1, BW2 and BW3 agree*.

Any of the three algorithms as well as the BW algorithm might have several positive equilibria depending on the observed data and the initial point. In general, one should expect coexistence of boundary and positive equilibria [37,39].

**Lemma 4.4.**

*The solution to the ODE system of BW3 exists for all times and any initial condition. Furthermore, assume* $(\theta^{\prime }(t),\psi^{\prime }(t))\to (\theta^{*},\psi^{*})\in \Theta_{0}$ *as* *t* → ∞ *for some initial condition, then* *x*(*t*) → x* *for some positive vector* x*, *where* *x*(*t*) *denotes the vector of concentrations of all species except the species* *θ*′, *ψ*′.

**Figure 3.**
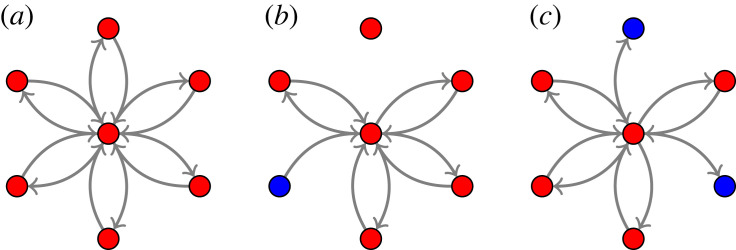
Each subnetwork in table 1 is a catalytic mono-molecular reaction network with time-dependent reaction rates. The designated species with indices h*, (h*, h*) or v*, depending on the subnetwork, correspond to the nodes in the middle of the reaction graphs. (*a*) If the reaction rates are all positive, then the reaction graph is strongly connected. (*b*) Three reaction rates are zero, breaking the graph into two connected components and creating one node (blue) from which all mass eventually disappears. (*c*) Two reaction rates are zero, creating two dead-end nodes (blue) from which mass cannot flow out. All mass eventually accumulates in the blue nodes.

If the initial point (*θ*_0_, *ψ*_0_) belongs to $\Theta \setminus \Theta_{0}$, then the dynamics of BW1/BW2 and BW3 might differ. Imagine that one or more of the (time-dependent) reaction rates are set to zero in figure 3. This could happen in different ways. In figure 3*b*, the graph is broken into two, effectively replacing one conserved quantity with two conserved quantities, one for each subgraph (one graph has only one node, the other six). The equilibrium depends on how much ‘mass’ is allocated to each subgraph initially. The same is the case if two dead-end nodes are created, nodes from which no mass can flow out; see figure 3*c*. Also here, an additional conserved quantity is created. See lemma A.3 for further details.

To complete the story, discrepancies between BW1 and BW2 (and similarly BW3) might also be found if some reaction rates eventually become zero. We illustrate this with a simple example. Imagine a two-species reaction network *X*_1_ → *X*_2_ with rate *κ*(*t*) = e^−*t*^, *t* ≥ 0, and conserved amount *T* = *x*_1_(*t*) + *x*_2_(*t*). It has solution *x*_1_(*t*) = *x*_1_(0)exp (e^−*t*^ − 1) → *x*_1_(0)/*e* as *t* → ∞, whereas the limiting reaction network with zero reaction rate has constant solution. Thus, a trajectory of the limiting reaction network only approximates a trajectory of the reaction network with time-dependent reaction rate, if the initial condition of the latter is *e* ≈ 2.71 times that of the former.

## 5. Multiple sequences and unknown *π*

If there are multiple observed sequences, *v*_1_, …, *v*_*R*_, $v_{i}=(v_{i1},\ldots ,v_{iL_{i}})$, *i* = 1, …, *R*, potentially of different length, then the forward and backward algorithms in the BW algorithm are replaced with *R* forward and backward algorithms, one for each sequence and initialized with the same values of (*θ*, *ψ*) and *π* [41]. Similarly, the E-step is replaced by *R* E-steps, one for each sequence. The main difference lies in the M-step, which is replaced by [41]

$$\pi_{g}^{\prime }=\frac{1}{R}\sum_{i=1}^{R}\gamma_{ig},\;\theta_{gh}^{\prime }=\frac{\sum_{i=1}^{R}\sum_{\ell =1}^{L_{i}-1}\xi_{i\ell gh}}{\sum_{i=1}^{R}\sum_{\ell =1}^{L_{i}-1}\sum_{\;f\in H}\xi_{i\ell gf}}$$

and

$$\psi_{hw}^{\prime }=\frac{\sum_{i=1}^{R}\sum_{\ell =1}^{L_{i}}\gamma_{i\ell h}\delta_{w,v_{i\ell }}}{\sum_{i=1}^{R}\sum_{\ell =1}^{L_{i}}\gamma_{i\ell h}},$$

where *π* now is updated and the additional index *i* in *γ*_*i*ℓ*h*_ and *ξ*_*i*ℓ*gh*_ refers to the *i*th sequence, and otherwise similar to the one sequence algorithm (if *π* is considered known, the update step for *π* is just ignored). The forward and backward algorithms and the E-step are implemented similarly to the one sequence reaction network with a set of species for each observed sequence and common species for *θ*, *ψ* and *π*. The M-step might be implemented by the reactions

5.1 $$\pi_{g}^{\prime }+\gamma_{i1g^{*}}\to \pi_{g^{*}}^{\prime }+\gamma_{i1g^{*}}$$

5.2 $$\pi_{g^{*}}^{\prime }+\gamma_{i1g}\to \pi_{g}^{\prime }+\gamma_{i1g}$$

$$\begin{array}{rl} \theta_{gh}^{\prime }+\xi_{i\ell gh^{*}} & \to \theta_{gh^{*}}^{\prime }+\xi_{i\ell gh^{*}}\\ \theta_{gh^{*}}^{\prime }+\xi_{i\ell gh} & \to \theta_{gh}^{\prime }+\xi_{i\ell gh}\\ \psi_{hw}^{\prime }+\gamma_{i\ell h}+E_{\ell v^{*}} & \to \psi_{hv^{*}}^{\prime }+\gamma_{i\ell h}+E_{\ell v^{*}}^{i}\\ \psi_{hv^{*}}^{\prime }+\gamma_{i\ell h}+E_{\ell w} & \to \psi_{hw}^{\prime }+\gamma_{i\ell h}+E_{\ell w}^{i}, \end{array}$$

where $E_{\ell w}^{i}$ is defined similarly to *E*_ℓ*w*_, but for the *i*th sequence alone. All these reactions take the same mono-molecular form as in the one sequence algorithm, resulting in statements analogous to theorems 4.1–4.3 (with analogous proofs). The reaction networks for the *R* sequences are executed simultaneously for the *R* sequences in the three different ways BW1, BW2 and BW3.

## 6. Examples

**Figure 4.**
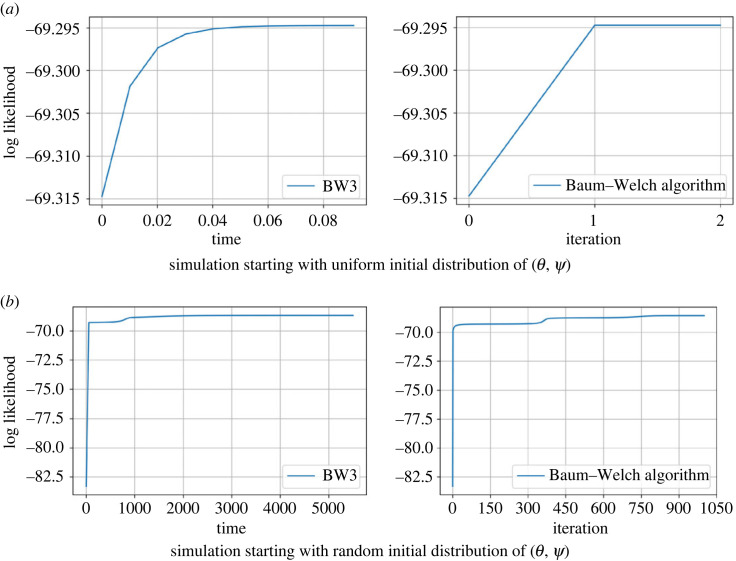
Dynamics of the BW3 and the BW algorithm for an HMM with two hidden states and two visible states for an observed sequence of length *L* = 100, and different initialization. (*a*) Log-likelihood of the sequence for BW3 (left) compared to BW algorithm (right) starting with equal initial parameter values. (*b*) Log-likelihood of the sequence for the BW3 (left) compared to BW algorithm (right) starting with random initial parameter values. In both cases, the likelihood is non-decreasing over time/iterations. See the main text and appendix A for details.

To illustrate the performance of BW3 versus the BW algorithm, we simulated an observed sequence of length *L* = 100 from an HMM with two hidden states and two visible states *v*_1_, *v*_2_; see appendix A for details and figure 4. Out of the 100 symbols, 49 were *v*_1_, while 51 were *v*_2_. Then, we ran the two algorithms for different choices of initial values with *π* = (0.5, 0.5) fixed. In one case, the two algorithms returned the same estimated values (figure 4*a*), namely

$$(\theta^{\prime },\psi^{\prime })=(\left[\begin{array}{cc} 0.5 & 0.5\\ 0.5 & 0.5 \end{array}\right],\left[\begin{array}{cc} 0.51 & 0.49\\ 0.51 & 0.49 \end{array}\right]),$$

far from the true values (see appendix A), while in the second case (figure 4*b*), the two algorithms returned markedly different values. For the BW algorithm, we obtained

$$(\theta^{\prime },\psi^{\prime })=(\left[\begin{array}{cc} 0.150 & 0.850\\ 0.998 & 0.002 \end{array}\right],\left[\begin{array}{cc} 0.642 & 0.358\\ 0.356 & 0.644 \end{array}\right]),$$

with log-likelihood −68.5617, while for BW3, we obtained values on the boundary,

$$(\theta^{\prime },\psi^{\prime })=(\left[\begin{array}{cc} 0.000 & 1.000\\ 0.229 & 0.771 \end{array}\right],\left[\begin{array}{cc} 0.000 & 1.000\\ 0.631 & 0.369 \end{array}\right]),$$

with log-likelihood −68.6750.

As a second example, we study a small observed sequence *v*_2_, *v*_1_, *v*_2_, *v*_1_, *v*_2_ of length 5, generated from an HMM with two hidden states and two visible states. With initial parameter values given in appendix A, the BW algorithm returns the boundary equilibrium

$$(\theta^{\prime },\psi^{\prime })=(\left[\begin{array}{cc} 0 & 1\\ 1 & 0 \end{array}\right],\left[\begin{array}{cc} 0 & 1\\ 1 & 0 \end{array}\right]),$$

while the BW3 returns a different boundary equilibrium

$$(\theta^{\prime },\psi^{\prime })=(\left[\begin{array}{cc} 0.366 & 0.634\\ 1 & 0 \end{array}\right],\left[\begin{array}{cc} 0 & 1\\ 1 & 0 \end{array}\right]).$$

Both examples illustrate that at the boundary different things might happen. In the latter case, the BW3 equilibrium belongs to Θ. However, when initiated at that point, the BW algorithm returns the first equilibrium point. Hence, the BW3 equilibrium is not an equilibrium of the BW algorithm (as the equilibrium in not positive, then theorem 4.3 is not contradicted).

**Figure 5.**
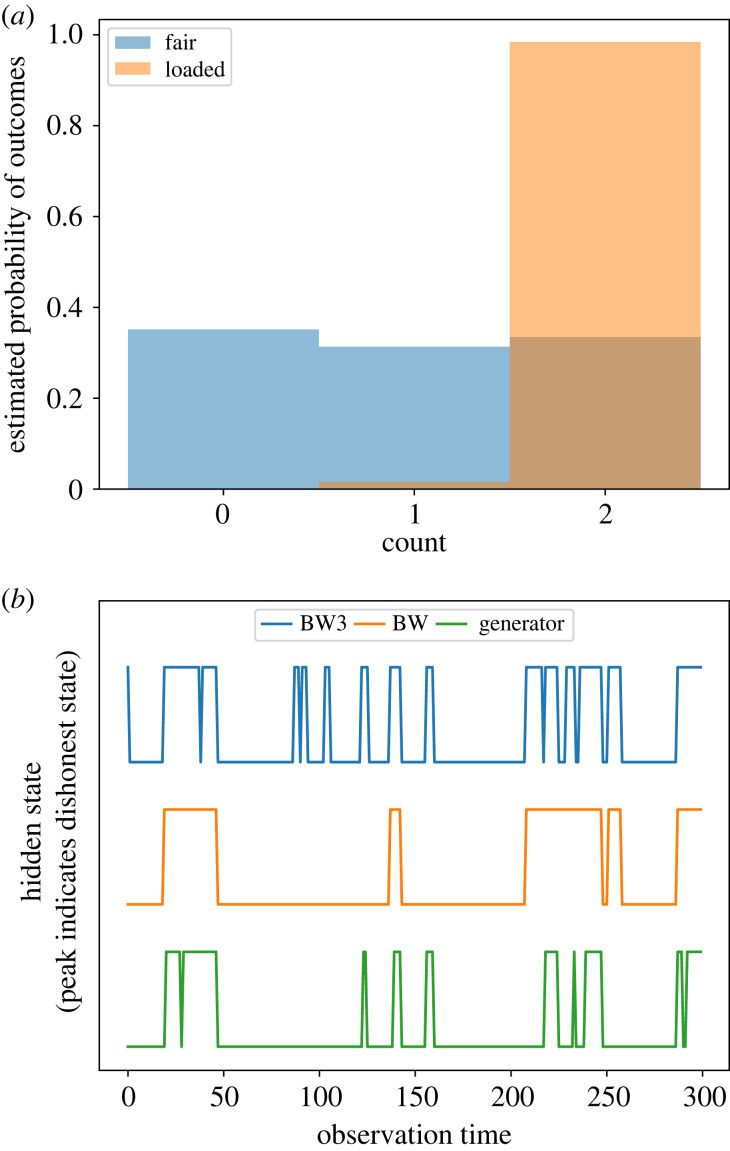
Detection of a dishonest casino. Inferred statistics in the dishonest casino example. (*a*) Outcome probabilities of the loaded and fair die estimated from the generator HMM for 300 die rolls. (*b*) Prediction of the hidden states on the test data (last 150 rolls) using the generator HMM and after training BW and BW3 on first 150 rolls with a random initial distribution of parameters.

We further study an example where the initial probabilities can be tuned by adding new reactions according to equations (5.1)–(5.2). Consider a casino game which depends on the output of a three-sided die. A fair die would have equal probabilities of each outcome [42]. However, the casino can switch to an unfair die with unequal outcome probabilities. The player only observes the die outcome sequence. An HMM with two hidden states of the casino (Honest and Dishonest) with three visible states as the outcome of the die can be used to predict if and when the casino is dishonest [42]. We use the following HMM:

$$(\pi^{\prime },\theta^{\prime },\psi^{\prime })=(\left[\begin{array}{c} 1.0\\ 0.0 \end{array}\right],\left[\begin{array}{cc} 0.95 & 0.05\\ 0.25 & 0.75 \end{array}\right],\left[\begin{array}{ccc} 0.34 & 0.33 & 0.33\\ 0.01 & 0.01 & 0.98 \end{array}\right])\;$$

to generate sample data with 300 rolls, where the Dishonest state has unequal probabilities of the die roll. The statistics of the generated sample is visualized in figure 5*a*. We then start with a random initial distribution of the HMM parameters and train it using both BW and BW3 on the training data (first 150 rolls) and infer the hidden states based on the learned parameters on the test data (next 150 rolls). BW converges to

$$(\pi^{\prime },\theta^{\prime },\psi^{\prime })=(\left[\begin{array}{c} 0.0\\ 1.0 \end{array}\right],\left[\begin{array}{cc} 0.9 & 0.01\\ 0.03 & 0.96 \end{array}\right],\left[\begin{array}{ccc} 0.03 & 0.07 & 0.90\\ 0.37 & 0.35 & 0.28 \end{array}\right]),$$

with a log-likelihood of −147.9. BW3 converges to

$$(\pi^{\prime },\theta^{\prime },\psi^{\prime })=(\left[\begin{array}{c} 0.81\\ 0.19 \end{array}\right],\left[\begin{array}{cc} 0.48 & 0.52\\ 0.43 & 0.57 \end{array}\right],\left[\begin{array}{ccc} 0.28 & 0.23 & 0.49\\ 0.27 & 0.35 & 0.38 \end{array}\right]),$$

with a log-likelihood of −157.4. The inference result of the training is visualized in figure 5*b*. Spikes indicate the switch to the hidden state Dishonest. We find that both BW and BW3 predict the hidden state sequence with high accuracy when compared with the HMM used for generating the sequences. More details can be found in appendix A.

## 7. Discussion

Some machine learning algorithms like gradient descent are based on continuous-time dynamical systems while others like message passing appear essentially discrete. Before our work, the BW algorithm has fallen in the second category. Performing the different steps in order was seen as an important part of the algorithm. Here, we have shown a continuous-time dynamical system based on the BW algorithm which implements HMM learning. Our work has exposed that there is a greater design space for BW algorithm implementations than was previously known. Specifically, the steps need not be run to completion, and need not be run in order. This reduces the synchronization burden for distributed implementations of such extended BW algorithms. As a result, our algorithm can be implemented on a distributed network of edge devices, and in this manner serves as a federated learning scheme. Our work exposes that reaction networks are a natural design language to think about the design of machine learning algorithms when the data and computation need to be handled in a distributed manner with minimum overhead of synchronization.

Our implementation of this algorithm is explicitly given in the form of a chemical reaction network. This opens the possibility of molecular implementations of this algorithm. If implemented in an artificial cell, it might provide the cell with the ability to sample possible realities, and act according to these imaginings. This is important because the world of molecules is a noisy world. To obtain exquisite control over a large number of molecules—which is one of the main goals of nanotechnology—requires algorithms that will be robust to such noise. Denoising or error correction algorithms are essentially statistical algorithms of the kind that we have implemented here. Thus, not only does our work point to future technological directions in nanotechnology, we believe reaction network implementations of algorithms of this kind are inevitable when attempting to exquisitely control large ensembles of molecules.

One of the big challenges in biology has been the immense complexity of living cells. While on one hand, cells are capable of behaviours of incredible sophistication, on the other hand our imagination about their workings has been limited to a very low level of abstraction described by systems biology. To understand the behaviour of cells will require the invention of layers of abstraction above that of systems biology. These layers will have to explain the algorithmic power of biochemical reaction networks. Biochemical reaction networks in living systems are known to be performing inference. There may be opportunity to understand them from the vantage point of our work which is giving concrete schemes by which reaction networks can lead to inference. Our design has reproduced certain characteristics found in biochemical reaction networks like the ubiquitous presence of enzymes and futile cycles, for example in phosphorylation and dephosphorylation of the same site on a protein. This reproduction has led to a novel idea to explain futile cycles: their purpose is to achieve probabilistic inference. For example, reactions of the form $A\to A^{*}$ catalysed in the forward direction by a kinase and in the backward direction by a phosphatase are shown here to be the paradigmatic form of reactions needed to carry out probabilistic inference. A research programme to pursue this idea to its logical conclusions is suggested by the work here, but is beyond the scope of the current paper.

## Data Availability

Code used for the simulation of examples in this paper may be found at: https://github.com/GeekyPeas/Chemical-Baum-Welch-Algorithm. The data are provided in electronic supplementary material [43].

## Appendix A

**A.1. The ODE system of the Baum–Welch reaction network**

With mass-action kinetics the ODE system of the reaction network in table 1 becomes

A 1 $${\dot{\alpha }}_{1h}=\alpha_{1h^{*}}\pi_{h}\psi_{hv_{1}}-\alpha_{1h}\pi_{h^{*}}\psi_{h^{*}v_{1}},$$

A 2 $${\dot{\alpha }}_{\ell h}=\alpha_{\ell h^{*}}\underset{g\in H}{\sum }\alpha_{\ell -1,g}\theta_{gh}\psi_{hv_{\ell }}-\alpha_{\ell h}\underset{g\in H}{\sum }\alpha_{\ell -1,g}\theta_{gh^{*}}\psi_{h^{*}v_{\ell }},$$

A 3 $${\dot{\beta }}_{L}=0,$$

A 4 $${\dot{\beta }}_{\ell h}=\beta_{\ell h^{*}}\underset{g\in H}{\sum }\beta_{\ell +1,g}\theta_{gh}\psi_{hv_{\ell +1}}-\beta_{\ell h}\underset{g\in H}{\sum }\beta_{\ell +1,g}\theta_{gh^{*}}\psi_{h^{*}v_{\ell +1}},$$

A 5 $${\dot{\gamma }}_{\ell h}=\gamma_{\ell h^{*}}\alpha_{\ell h}\beta_{\ell h}-\gamma_{\ell h}\alpha_{\ell h^{*}}\beta_{\ell h^{*}},$$

A 6 $$\begin{array}{rl} & {\dot{\xi }}_{\ell gh}=\xi_{\ell h^{*}h^{*}}\alpha_{\ell g}\theta_{gh}\beta_{\ell +1,g}\psi_{hv_{\ell +1}}\\ & \;-\xi_{\ell gh}\alpha_{\ell h^{*}}\theta_{h^{*}h^{*}}\beta_{\ell +1,h^{*}}\psi_{h^{*}v_{\ell +1}}, \end{array}$$

A 7 $${\dot{\theta }}_{gh}^{\prime }=\theta_{gh^{*}}^{\prime }\overset{L-1}{\underset{\ell =1}{\sum }}\xi_{\ell gh}-\theta_{gh}^{\prime }\overset{L-1}{\underset{\ell =1}{\sum }}\xi_{\ell gh^{*}}$$

A 8 $$\mathrm{and}\;{\dot{\psi }}_{hw}^{\prime }=\psi_{hv^{*}}^{\prime }\overset{L}{\underset{\ell =1}{\sum }}\gamma_{\ell h}\delta_{w,v_{\ell }}-\psi_{hw}^{\prime }\overset{L}{\underset{\ell =1}{\sum }}\gamma_{\ell h}\delta_{v^{*},v_{\ell }},$$

where *h** ∈ *H* and *v** ∈ *V* are fixed states, $h\neq h^{*}$ in (A 1)–(A 5), $\left(g,h)\neq (h^{*},h^{*}\right)$ in (A 6), *g* ∈ *H*, $h\neq h^{*}$ in (A 7), and *h* ∈ *H*, $w\neq v^{*}$ in (A 8). In (A 2), ℓ = 2, …, *L*, in (A 4) and (A 6), ℓ = 1, …, *L* − 1, and in (A 5), ℓ = 1, …, *L*. The equations for the remaining species are obtained as ${\dot{\alpha }}_{1h^{*}}=-\sum_{h\neq h^{*}}{\dot{\alpha }}_{1h}$, and similarly for the other species, reflecting that the sum $A_{\ell }=\sum_{h}\alpha_{1h}(t)$ is conserved through time. The explicit dependence on time is suppressed in (A 1)–(A 8). The species *E*_ℓ*w*_ are left out for convenience.

**A.2. Reformulation of the Baum–Welch algorithm**

The BW algorithm can be formulated in an equivalent way that exposes the relationship to the dynamics of the BW reaction network; see the electronic supplementary material for proof and details. Define *α*_ℓ*h*_, *β*_ℓ*h*_, *h* ∈ *H*, ℓ = 1, …, *L*, recursively by

$$\alpha_{1h}=\frac{A_{1}\pi_{h}\psi_{hv_{1}}}{\sum_{g\in H}\pi_{g}\psi_{gv_{1}}},\;\alpha_{\ell h}=\frac{A_{\ell }\sum_{g\in H}\alpha_{\ell -1,g}\theta_{gh}\psi_{hv_{\ell }}}{\sum_{\;f\in H}\sum_{g\in H}\alpha_{\ell -1,f}\theta_{\;fg}\psi_{gv_{\ell }}}$$

and

$$\beta_{Lh}=\frac{B_{L}}{\#H},\;\beta_{\ell h}=\frac{B_{\ell }\sum_{g\in H}\theta_{hg}\psi_{gv_{\ell +1}}\beta_{\ell +1,g}}{\sum_{\;f\in H}\sum_{g\in H}\theta_{\;fg}\psi_{gv_{\ell +1}}\beta_{\ell +1,g}},$$

where *A*_ℓ_, *B*_ℓ_ are arbitrary positive constants, ℓ = 1, …, *L*. These two recursions correspond to the forward and backward algorithms of the standard BW algorithm, respectively, with the essential difference being that *α*_ℓ*h*_, *β*_ℓ*h*_ scale to *A*_ℓ_, *B*_ℓ_, respectively. The E-step and the M-step are identical to the corresponding steps of the standard BW algorithm.

In this formulation, *α*_ℓ*h*_, *β*_ℓ*h*_, *h* ∈ *H*, in the BW algorithm can be seen as the equilibrium values of the corresponding reaction networks, $R_{\ell }^{\alpha },R_{\ell }^{\beta }$; see lemma A.3. As the sum of *γ*_ℓ*h*_, *ξ*_ℓ*g h*_, *θ*_ℓ*h*_, respectively, over *h* is one, a similar interpretation applies in this case. Similarly for *ψ*_ℓ*v*_, where the sum over *v* is one.

**A.3. Mono-molecular reaction networks**

Properties of mono-molecular reaction networks are of particular importance for the proofs of the statements in the main text.

Consider a mono-molecular reaction network with *n* species, *X*_1_, …, *X*_*n*_, and reactions

$$X_{r_{i}}\overset{\kappa_{i}(t)}{\to }X_{\;p_{j}},\;\text{for}\;i=1,\ldots ,m,$$

where $r_{i}\neq p_{i}$, *r*_*i*_, *p*_*i*_ ∈ {1, …, *n*} and *κ*_*i*_(*t*), *i* = 1, …, *m*, are mass-action time-dependent reaction rate constants, *t* ≥ 0. We assume *κ*_*i*_(*t*) > 0 and let *κ*(*t*) = (*κ*_1_(*t*), …, *κ*_*m*_(*t*)) be the vector of reaction rate constants. Furthermore, we assume there is at most one reaction *i* such that (*r*_*i*_, *p*_*i*_) = (*r*, *p*) ∈ {1, …, *n*}^2^. (If there is more than one reaction, the reaction rate constants are summed.)

The (non-autonomous) ODE system of the reaction network is

$${\dot{x}}_{i}=-x_{i}\;\underset{\;j\;:\;r_{j}=i}{\sum }\kappa_{j}(t)+\underset{\;j\;:\;p_{j}=i}{\sum }\kappa_{j}(t)x_{\;p_{j}},\;i=1,\ldots ,n.$$

Define the *n* × *n* matrix *A*(*t*) = (*a*_*ij*_(*t*))_*i*,*j*=1, …,*n*_ by

$$a_{ij}(t)=\kappa_{k}(t),\;\text{if there is }k\in \{1,\ldots ,m\}\;\text{such that}\;(r_{k},p_{k})=(j,i)$$

and

$$a_{ii}(t)=-\underset{\;j\;:\;r_{j}=i}{\sum }\kappa_{j}(t).$$

Then, the ODE system can be written as

A 9 $$\dot{x}=A(t)x.$$

The matrix *A*(*t*) is a Laplacian matrix with column sums zero, the off-diagonal elements are non-negative and the diagonal elements are non-positive. In particular, the non-zero eigenvalues of *A*(*t*) have negative real part [44,45].

The Laplacian graph *G* corresponding to *A*(*t*) is independent of time as *κ*(*t*) is positive for *t* ≥ 0 by assumption. Let *λ* be the number of connected components of *G* and *τ* the number of maximal strongly connected subgraphs. Always *τ* ≥ *λ*. In figure 3*a*: *τ* = *λ* = 1, figure 3*b*: *τ* = *λ* = 2, and figure 3*c*: *τ* = 2, *λ* = 1. Each connected component of the Laplacian graph gives rise to a conserved quantity, namely the sum of the variables in the connected component:

$$T_{j}=\underset{i\in G_{j}}{\sum }x_{i},\;T_{j}\geq 0,$$

where *G*_*j*_ is the *j*th connected component, *j* = 1, …, *λ*. The non-negative vector $T=(T_{1},\ldots ,T_{\lambda })$ defines an invariant subspace of (A 9). Let $z_{j}\in R^{n}$ be the vector with *i*th component 1 if *i* ∈ *G*_*j*_ and otherwise zero. Then, *z*_*j*_ is a left kernel vector of *A*(*t*), *z*_*j*_*A*(*t*) = 0, and a linear first integral of (A 9).

Lemma A.1. *The following hold*:

i *The rank of* *A*(*t*) *equals* *n* − *λ* *if and only if* *τ* = *λ*.
ii *If* *τ* = *λ*, *then* $z_{1},\ldots ,z_{\lambda }$ *is a basis of the left kernel of* *A*(*t*). *Any linear first integral is a linear combination of* $z_{1},\ldots ,z_{\lambda }$.
iii *Assume* *A*(*t*) = *A* *is independent of time. If* *τ* = *λ*, *then there is a unique non-negative exponentially and globally stable equilibrium within each invariant subspace given by* *T* ≥ 0. *The equilibrium is positive if and only if the connected components are strongly connected and* *T* > 0.

Lemma A.2. *Assume* *τ* = *λ* *and that* *κ*(*t*), *t* ≥ 0, *converges exponentially fast towards* $\kappa =(\kappa_{1},\ldots ,\kappa_{m})\in R_{>0}^{m}$ *as* *t* → ∞, *i.e. there exists* *γ*_1_ > 0 *and* *K*_1_ > 0 *such that*

$$\|\kappa (t)-\kappa \|\leq K_{1}e^{-\gamma_{1}t}\;\text{for}\;t\geq 0.$$

*Let* *A* *be the matrix* *A*(*t*) *with* *κ* *inserted for* *κ*(*t*). *Then, any solution to* $\dot{x}=A(t)x$ *converges exponentially fast towards the unique globally stable equilibrium* $x^{*}\in R_{>0}^{n}$ *of the ODE system* $\dot{x}=Ax$ *within the relevant invariant subspace, provided* *T* > 0. *That is, there exists* *γ*_2_ > 0 *and* *K*_2_ > 0 *such that*

$$\|x(t)-x^{*}\|\leq K_{2}e^{-\gamma_{2}t}\;\text{for}\;t\geq 0.$$

Lemma A.3. *Consider a mono-molecular reaction network with reactions*

$$X_{i}\overset{\kappa_{n}}{\underset{\kappa_{i}}{⇌}}X_{n},\;\text{for}\;i=1,\ldots ,n-1,$$

*such that* *κ*_*i*_ > 0 *for* *i* = 1, …, *n* − 1 (*then the Laplacian graph is as in figure 3a*)*. In particular*, *λ* = *t* = 1. *Then, the unique positive equilibrium for* *T* > 0 *is*

$$x^{*}=(x_{1}^{*},\ldots ,x_{n}^{*})\;\mathrm{and}\;x_{i}^{*}=\frac{T\kappa_{i}}{\sum_{\;j=1}^{n}\kappa_{j}},\;\text{for}\;i=1,\ldots ,n.$$

**A.4. Proofs**

We provide proofs of theorems 4.1–4.3 and lemma 4.4. The remaining statements are proven in the electronic supplementary material, except lemma A.3 which is straightforward. We assume *π* > 0, *L* ≥ 2, and note that similar arguments apply if *π* ≥ 0 and *L* ≥ 3.

**A.4.1. Proof of theorem 4.1**

It is sufficient to demonstrate that (*θ*_*n*_, *ψ*_*n*_), *n* ≥ 0, are the same and positive after each iteration of the BW algorithm and BW1. For this, assume *θ*, *ψ* (and *π*) are positive. Then the subnetwork $R_{1}^{\alpha }$ is mono-molecular with species *α*_1*h*_, *h* ∈ *H*, and fixed positive reaction rates. The Laplacian graph is strongly connected and *τ* = *λ* = 1. Lemma A.1(iii) gives global stability of the unique positive equilibrium for the concentrations of the species *α*_1*h*_, *h* ∈ *H*, identical to that of the BW algorithm (compare lemma A.3). This argument is repeated inductively for each subnetwork (in the order listed for BW1). Specifically, for the *k*th subnetwork (*k* = 2, …, 4*L*), the equilibrium of the *k*′th subnetwork, *k*′ < *k*, is positive by induction hypothesis. Hence the reaction rates for the *k*th subnetwork are positive and fixed, given by the equilibrium values of the subnetworks *k*′ < *k* and *θ*, *ψ*. Hence, the Laplacian graph is strongly connected with *τ* = *λ* = 1. Using lemma A.1(iii) completes the argument. In particular, (*θ*′, *ψ*′) is positive and the same for the BW algorithm and BW1.

**A.4.2. Proof of theorem 4.2**

The proof is similar to that of theorem 4.1. The layered structure of BW2 implies that the equilibrium of the species in the *k*th subnetwork is unaffected by the presence of the *k*′th subnetwork, *k*′ > *k*. Assume *θ*, *ψ* (and *π*) are positive. Then the subnetwork $R_{1}^{\alpha }$ is mono-molecular with species *α*_1*h*_, *h* ∈ *H*, and fixed positive reaction rates. The Laplacian graph is strongly connected and *τ* = *λ* = 1. Lemma A.1(iii) gives exponential stability of the unique positive equilibrium for the concentrations of the species *α*_1*h*_, *h* ∈ *H*. By lemma A.1(iii), it is the same as that of BW1. Consider next the subnetwork $R_{2}^{\alpha }$. It is mono-molecular with positive time-dependent reaction rates, given by the concentrations of the species *α*_1*h*_, *h* ∈ *H*, at time *t* ≥ 0 and *θ*, *ψ*. Hence, the Laplacian graph is strongly connected and *τ* = *λ* = 1. Applying lemma A.2 yields exponential convergence towards the unique positive equilibrium for the concentrations of the species *α*_2*h*_, *h* ∈ *H*. This equilibrium is the same as that of BW1 for $R_{2}^{\alpha }$ (lemma A.2). The theorem now follows by repeated application of the above procedure for all subnetworks.

**A.4.3. Proof of theorem 4.3**

If the catalyst species concentrations of a subnetwork at equilibrium are positive, then the unique equilibrium concentrations of the non-catalyst species are likewise positive (lemma A.3). Consequently, if *θ*, *ψ* (and *π*) are positive, then the unique equilibrium concentrations of the species *α*_1 *h*_, *h* ∈ *H*, in $R_{1}^{\alpha }$ are positive. Inductively, the equilibrium concentrations in any subnetwork are positive, if *θ*, *ψ* (and *π*) are positive.

For BW1 and BW2, if $(\theta ,\psi )\in \Theta_{0}$ is an equilibrium, then (*θ*′, *ψ*′) is well-defined and (*θ*′, *ψ*′) = (*θ*, *ψ*). Consequently, the species concentrations at equilibrium solve the same equations in the three cases, thus the sets of equilibria are the same. The same reasoning gives the uniqueness statement. Theorem 4.1 gives uniqueness for the BW algorithm.

**A.4.4. Proof of lemma 4.4**

The first part follows by standard arguments as the trajectories are confined to a compact space. The second part follows from [46, theorem 1.2] and lemma A.1(iii).

**A.5. Simulated examples**

We consider an HMM with two hidden states and two visible states *v*_1_, *v*_2_, and generate a random sequence of length *L* = 100 from *π* = (0.5, 0.5) (fixed throughout) and

$$(\theta ,\psi )=(\left[\begin{array}{cc} 0.2 & 0.8\\ 0.7 & 0.3 \end{array}\right],\left[\begin{array}{cc} 0.75 & 0.25\\ 0.3 & 0.7 \end{array}\right]).$$

The generated sequence is

$$\begin{array}{rl} & (v_{2},v_{1},v_{2},v_{2},v_{1},v_{2},v_{2},v_{1},v_{2},v_{1},v_{1},v_{1},v_{2},v_{1},v_{1},v_{1},v_{2},\\ & \;v_{1},v_{1},v_{2},v_{2},v_{2},v_{2},v_{1},v_{1},v_{2},v_{2},v_{1},v_{1},v_{1},v_{2},v_{1},v_{2},v_{2},\\ & \;v_{2},v_{2},v_{1},v_{1},v_{1},v_{2},v_{2},v_{2},v_{1},v_{2},v_{1},v_{2},v_{1},v_{1},v_{2},v_{2},v_{2},\\ & \;\;v_{2},v_{1},v_{1},v_{1},v_{1},v_{2},v_{1},v_{2},v_{1},v_{2},v_{1},v_{1},v_{1},v_{1},v_{1},v_{1},v_{2},\\ & \;\;v_{1},v_{2},v_{2},v_{1},v_{2},v_{1},v_{2},v_{1},v_{2},v_{1},v_{2},v_{1},v_{2},v_{1},v_{1},v_{2},v_{2},\\ & \;\;\;v_{2},v_{2},v_{1},v_{2},v_{2},v_{1},v_{1},v_{1},v_{1},v_{1},v_{1},v_{2},v_{2},v_{2},v_{2}). \end{array}$$

The log-likelihood of the sequence is −68.7955. We trained the HMM using BW3 and the BW algorithm, starting from two different initials values: (1) equal transition and emission probabilities

$$(\theta^{\prime },\psi^{\prime })=(\left[\begin{array}{cc} 0.5 & 0.5\\ 0.5 & 0.5 \end{array}\right],\left[\begin{array}{cc} 0.5 & 0.5\\ 0.5 & 0.5 \end{array}\right])\;$$

and (2) randomly chosen values

$$(\theta^{\prime },\psi^{\prime })=(\left[\begin{array}{cc} 0.774 & 0.226\\ 0.427 & 0.573 \end{array}\right],\left[\begin{array}{cc} 0.916 & 0.084\\ 0.510 & 0.490 \end{array}\right]).\;$$

The other species in the reactions of the BW3 were initialized with (1) equal concentrations and (2) concentrations obtained from one step of the BW algorithm and appropriate normalization.

In the second example, the initial values are *π* = (0.6, 0.4) (fixed), and initial transition and emission probabilities

$$(\theta^{\prime },\psi^{\prime })=(\left[\begin{array}{cc} 0.7 & 0.3\\ 0.4 & 0.6 \end{array}\right],\left[\begin{array}{cc} 0.6 & 0.4\\ 0.6 & 0.4 \end{array}\right]).$$

In the final example of a dishonest casino, the randomly chosen initial values are *π* = (0.83, 0.17), and initial transition and emission probabilities

$$(\theta^{\prime },\psi^{\prime })=(\left[\begin{array}{cc} 0.1 & 0.9\\ 0.02 & 0.98 \end{array}\right],\left[\begin{array}{ccc} 0.12 & 0.08 & 0.80\\ 0.07 & 0.89 & 0.04 \end{array}\right]).$$

For all of the examples, BW3 was simulated using the ODEINT package from the Scipy library for a resolution of 100 points starting with time 0 to the final time. For the BW algorithm, the hmmlearn package was used from the Scikit library.
